# Water and other volatiles on Mars

**DOI:** 10.1093/nsr/nwae094

**Published:** 2024-03-12

**Authors:** Sen Hu, Yubing Gao, Zhan Zhou, Liang Gao, Yangting Lin

**Affiliations:** Key Laboratory of the Earth and Planetary Physics, Institute of Geology and Geophysics, Chinese Academy of Sciences, China; Key Laboratory of the Earth and Planetary Physics, Institute of Geology and Geophysics, Chinese Academy of Sciences, China; College of Earth and Planetary Sciences, University of Chinese Academy of Sciences, China; Key Laboratory of the Earth and Planetary Physics, Institute of Geology and Geophysics, Chinese Academy of Sciences, China; College of Earth and Planetary Sciences, University of Chinese Academy of Sciences, China; Key Laboratory of the Earth and Planetary Physics, Institute of Geology and Geophysics, Chinese Academy of Sciences, China; College of Earth and Planetary Sciences, University of Chinese Academy of Sciences, China; Key Laboratory of the Earth and Planetary Physics, Institute of Geology and Geophysics, Chinese Academy of Sciences, China

## Abstract

This perspective reviews the recent advances in martian water and other volatiles and addresses the associated scientific questions for future martian exploration missions.

Mars, the intriguing red planet, is one of the planets most similar to the Earth in our solar system. It is widely accepted that Mars could have had a warm and wet climate in its early history supported by numerous lines of evidence, e.g. topography, canyons, outflow channels, presence of phyllosilicates and evaporative salts, sedimentary formation, and many others (e.g. [1]), and thus Mars is believed to once have a potentially habitable environment which could be able to supply fundamental conditions for the evolution of potential lives. Therefore, discovery of biosignatures of potential life on Mars is becoming the top scientific objective in planetary sciences, boosting the continuous martian exploration missions over the world. However, present-day Mars is a cold and arid planet, indicating that Mars has experienced a notable climate change in its geologic history. The studies of potentially habitable to unhabitable paleoclimate transition of Mars place crucial constraints on potential lives, and also shed more light on understanding the habitable evolution history of the Earth. In this perspective, we review the recent advances in martian volatiles and address the associated scientific questions for future martian exploration missions.

The wet to arid paleoclimate change that occurred on Mars is directly coupled with the loss of martian surface water due to the interaction of solar wind with the martian atmosphere [2]. Water escape on Mars led to the hydrogen (H) isotopic composition of Mars’ atmosphere becoming progressively enriched in deuterium (D) by the faster escape rate of H than D [3], resulting in the present martian atmosphere being highly enriched in D/H ratio (δD up to ∼ 7000‰) [4]. Therefore, in turn, the evolution of water on Mars is a good proxy to unravel the paleoclimate evolution history of the planet. Moreover, the evolution of water and other volatiles on Mars is also critical to understand the geologic evolution history of Mars and thus has received particular attention in martian sciences. On another aspect, noble gases trapped in martian samples, such as Ar, Kr, Xe, and Ne, are also quite sensitive to martian atmospheric evolution [5].

There are at least four water/volatile reservoirs on Mars: atmosphere, polar ice cap and subsurface glaciers, crustal reservoir, and mantle reservoir, displaying contrasting characteristics in abundance and isotopic composition (Fig. 1). Volatiles in the martian atmosphere are better constrained than those of the other reservoirs since the measurements of the Viking space probe and has been advanced by the Curiosity rover, MAVEN orbiter, and Perseverance rover. The atmosphere of Mars is mainly composed of CO_2_ (95%) with minor N_2_ (2.8%), Ar (2%), and water vapor (0.03%) and displays heavier isotopes of H (δD ∼ 5000‰ ± 1080‰) [4], C (δ^13^C ∼ 46‰ ± 4‰) [6], N (δ^15^N ∼ 620‰ ± 160‰) [7], and Ar (δ^40^Ar ∼ 5360‰ ± 1000‰) [6] compared with those of Earth because of isotope fractionation via atmospheric escape caused by solar wind bombardment under a weak magnetic field [2]. The polar ice cap on Mars is mainly composed of CO_2_ ice, water ice, and dust particles in layers and displays seasonal variations in appearance. The water stored in the polar ice cap is estimated to be ∼ 20 m global equivalent layer (GEL) of water. Polar ice on Mars is likely the frozen martian atmosphere and acts as a buffer for sustaining the atmosphere cycling on Mars by sublimation and condensation [8].

**Figure 1.**
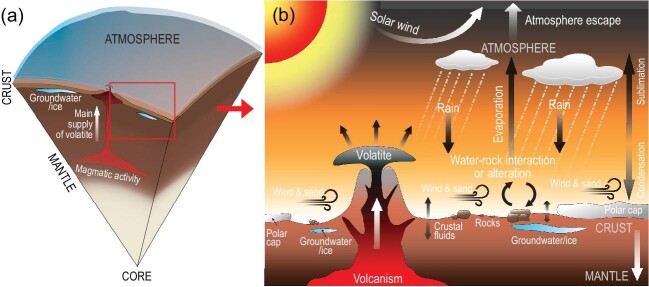
(a) Illustration of martian volcanism volatile transports from mantle source to surface. (b) Schematic illustration of wind, water-rock interaction, outgassing of volcanism, polar ice sublimation and condensation, and solar wind-induced volatile escape, which occurred on the surface of Mars.

The crust reservoir is relatively poorly constrained for water and volatiles compared with the atmosphere because various events happened in/on this venue. *In situ* measurements of the sedimentary rocks in the Gale crater have shown that they have elevated isotopic compositions of H, C, and N similar to the present martian atmosphere (e.g. [6]). These rocks could have been partially equilibrated with the atmosphere in isotopic compositions of volatile elements during emplacement and post-emplacement geological events at the near surface of Mars. The martian crust on average is estimated to contain ∼ 1410 ppm water, 450 ppm chlorine, and 106 ppm fluorine using known crust-mantle distributions for incompatible lithophile elements [9]. On the other hand, China's first martian mission, Tianwen-1, has shown that Mars could have modern water on its surface (e.g. [10]). Martian crust, especially the martian surface, displays high diversity in morphology, rock type, and lithology. The sedimentary rocks, clays, phyllosilicates, and alteration phases associated with water may be able to preserve the partial biosignatures of potential life and has been treated as the major target for exploration of this potential.

The intensive volcanic eruptions could have supplied adequate water and other volatiles to form an ancient ocean on early Mars (Fig. 1a). This ancient ocean is estimated to have had a depth ∼ 137 m GEL of water based on the D/H ratio of the present martian atmosphere and by the evolution physics of water on other planets [11]. Therefore, the martian mantle reservoir should have had higher water and volatile abundances in earlier times than the present-day value, but is poorly constrained because of the lack of pristine ancient martian rocks in hand so far. Martian basaltic meteorites, the igneous rocks derived from the martian mantle, are mostly younger than 700 Ma and are classified into enriched and depleted geochemical groups. The enriched mantle source was estimated to have 36–73 ppm H_2_O, slightly higher than the depleted source (14–23 ppm H_2_O), based on the measured H_2_O content of apatite, amphibole, and melt inclusions in martian meteorites [9]. However, the apatite, amphibole, and melt inclusions from various martian meteorites have elevated D/H ratios compared with the initial composition (terrestrial like), likely reflecting assimilation of crustal materials and post-emplacement water-rock interaction [12]. If we correct the contribution from the later event based on the D/H ratios, the martian mantle would be estimated to only have several ppm H_2_O for samples younger than 700 Ma [13]. The mantle reservoir has been proposed to be enriched in Cl (0.3 wt%) which plays a similar role as H_2_O in magmatic processes [14]; however, recent studies have shown that Cl in impact glasses and melt inclusion glasses from martian meteorites have received a contribution from crustal/surface Cl-rich fluid [13,15], yielding a Cl content of 4 ppm of the mantle reservoir after correction for crustal Cl contribution. Sulfur content in the mantle source could have not reached a saturation line in martian fugacity conditions, and was estimated to be <15 ppm [13]. H_2_O, Cl, and S contents in the martian mantle reservoirs of the young martian meteorites could be significantly lower than those of Earth. Carbon and N contents in the mantle reservoir are not well constrained. The growing *in situ* analysis of volatile content and its isotopic compositions point to the fact that most martian meteorites have been notably affected by the water-rock interaction at the surface of Mars before being ejected by asteroid impacts. Thus, understanding the water and volatile abundance in the martian mantle and its evolution over time is crucial in order to unravel the climate evolution history of Mars but is an extremely difficult task because of the deficit of ancient samples and their complex geologic processes (degassing, assimilation, and post-emplacement water-rock interaction).

One of the recent advances in martian volatiles is the identification of organics on Mars and in martian meteorites. Seasonal methane release and diverse organic matter (thiophenic, aromatic, and aliphatic compounds) were detected in Gale crater by the instruments on board the Curiosity rover [16,17], enhancing the possibility of potential life on Mars. Diverse organic molecules occurring within minerals have been detected by the Perseverance rover in Jezero crater [18], probably associated with the aqueous processes which may have played a key role in organic synthesis, transport, and preservation [19]. Organic matter was also identified in martian meteorites during the past decade. The newest fallen martian meteorite, Tissint, has abundant insoluble C-rich materials occurring along the cracks of the host rock or partially transformed into graphite and diamond in the shock-induced veins, indicating that an organic-rich fluid rock interaction could have occurred to facilitate the transportation of organics at the near surface of Mars [20]. A recent study has shown that Tissint has complex soluble organic matters which are similar to the ones identified in carbonaceous chondrites, indicating that some organics on Mars may have been delivered by asteroid impacts [21]. Currently, there is no concise conclusion that the organics identified on Mars and in martian meteorites have a biotic and indigenous origin, while the aqueous environment of ancient Mars would be favorable to record the biosignatures of potential life.

In summary, volatiles are important clues to understand the evolution history of the paleoclimate, potential life, habitable environment, and geology of Mars. New findings in the past decade strongly indicate that the red planet is more intriguing than we thought. The Perseverance rover is collecting the samples on Mars, which are scheduled to return to Earth by 2030. The new samples returned from Mars will supply ground truths for answering the unsolved questions in martian sciences, for instance, the existence of potential life, characteristics of volatile inventories, water-rock interaction, and many other topics. China's first martian sample return mission, Tianwen-3, is also under consideration. In the aspect of volatiles, we propose that Tianwen-3 should consider bringing ancient volcanic rocks/clasts and sedimentary rocks back to Earth for constraining the spatiotemporal evolution history of volatiles and investigating the potential for life on Mars.
